# Antioxidant Profiling of *Glycosmis cyanocarpa*: In Vitro Assays and Pearson’s Correlation Study

**DOI:** 10.1155/bmri/4377026

**Published:** 2026-08-20

**Authors:** Sania Ashrafi, Sadia Tasnim Mina, Raihana Haque Jebin, Firoj Ahmed

**Affiliations:** ^1^ Department of Pharmaceutical Chemistry, Faculty of Pharmacy, University of Dhaka, Dhaka, Bangladesh, du.ac.bd; ^2^ Department of Pharmacy, Faculty of Pharmacy, University of Dhaka, Dhaka, Bangladesh, du.ac.bd

**Keywords:** antioxidant, free radical scavenging, *Glycosmis cyanocarpa*, oxidative stress, Pearson′s correlation, total flavonoids, total phenolics

## Abstract

Oxidative stress is a major contributor to the development of various chronic illnesses, making the evaluation of plant‐derived antioxidants a crucial step in the search for novel therapeutic agents. *Glycosmis cyanocarpa* (Blume) Spreng., a relatively underexplored species native to South and Southeast Asia, has shown potential as a natural antioxidant source. Therefore, the current study aimed to assess the antioxidant potential of the crude methanolic extract of *G. cyanocarpa* (GCM) by determining its total phenolic content (TPC), total flavonoid content (TFC), evaluating its free radical scavenging and reducing power through DPPH, ABTS, and FRAP assays, and conducting Pearson’s correlation analysis to explore the associations among these parameters. The extract exhibited a TPC of 44.84 ± 0.99 mg gallic acid equivalent/g and a TFC of 60.83 ± 2.60 mg quercetin equivalent/g. GCM demonstrated significant antioxidant activity with IC_50_ values of 18.80 ± 0.42 *μ*g/mL in DPPH and 74.54 ± 0.31 *μ*g/mL in ABTS assays, and a ferric reducing capacity of 2.42 ± 0.33 mmol Fe^2+^ equivalent/g in the FRAP assay. Pearson correlation analysis revealed strong and positive correlations between DPPH and TPC (r = 0.998, *p* < 0.05) and FRAP with TFC (r = 0.999, *p* < 0.05) and indicated a significant association between phenolic and flavonoid compounds and antioxidant activity. ABTS values were also strongly correlated to TPC (r = 0.957) and TFC (r = 0.977). These findings highlight *G. cyanocarpa* as a potential source of natural antioxidants and recommend further work to isolate active compounds and explore their therapeutic importance in conditions associated with oxidative stress.

## 1. Introduction

Natural compounds are currently viewed as crucial new drug leads. Because of their structural diversity and biological activity profile, they are a good source for the lead identification for drug discovery and facilitate the design of novel drugs for treating a wide range of diseases. They can have synergistic activity [1], exhibit less side effects compared with synthetic counterparts [2], and selectively target cells to curtail the damage and total toxicity [3]. The advancement in recent analytical and computational platforms made it easier to examine natural compounds. Molecular docking, QSAR modeling, and machine learning, among other advanced technologies, aid researchers in the prediction of the possible targets and physiological activities of the natural products. In this regard, natural bioactive compounds are gaining fame as potential drugs.

*Glycosmis* is a genus in the Rutaceae family with 51 accepted species and 22 varieties [4]. It is an ample source of various secondary metabolites such as acridone‐, carbazole‐, quinolone‐, quinazoline‐alkaloids, flavonoids [5], phenolic glycosides, quinones, terpenoids, glycerides [6], gums, reducing sugars, tannins, and saponins [7] that are isolated from different parts of *Glycosmis* plants. The traditional uses of *Glycosmis* species include the treatment of intestinal worm infections, cancer, liver problems, fever, cough, rheumatism, anemia, skin disorders, wounds, and facial inflammation [4].

*Glycosmis cyanocarpa* (Blume) Spreng. is a less explored species of this genus. This is a small flowering tree or shrub that has longer than wide, oblong, ellipsoid, or ovoid‐shaped fruits and 4‐merous flowers with eight stamens and four‐locular ovaries. The plant grows in Bangladesh, Burma, Sri Lanka, Thailand, Malaysia, the Philippines, India, Nepal, South Tibet, and Indonesia (western) [6].

There have been a few publications on the phytochemical investigation of *G. cyanocarpa* in the past. Several significant bioactive substances identified and isolated from *G. cyanocarpa* include laipol and isolaipol [8], methoxyphenol, indole, stigmasterol, *β*‐sitosterol [9], penangin, *β*‐Caryophyllene oxide, and phytol [7].

Numerous authors looked into the plant’s antifungal [10], antimicrobial, cytotoxic, thrombolytic [9], and antioxidant [7] properties. Few studies have looked into the varied therapeutic activities of phytochemicals; most previous research focused on identifying, isolating, and assessing their bioactivity [11].

However, studies on other *Glycosmis* species have demonstrated the extracts’ potential antioxidant properties. *Glycosmis pentaphylla* demonstrates that it has strong antioxidant qualities in addition to its cytotoxic and antidiabetic effects [12]. Another study reported that the methanolic extract of *G. pentaphylla* significantly attenuates CCl_4_‐induced hepatic fibrosis by inhibiting TGF‐*β*/Smad signaling, reducing oxidative stress, and modulating inflammatory responses [13]. According to a different study assessing the methanolic extract of *G. pentaphylla*’s capacity to scavenge free radicals, the leaves exhibit very little antioxidant activity, whereas the stems show moderate levels of it [14]. The ethanolic extract of *G. pentaphylla* leaf exhibits potent antioxidant activity when compared with stem and root extracts [15]. Selenium nanoparticles synthesized using *G. pentaphylla* aqueous leaf extract also demonstrate great biocompatibility as well as negligible toxicity, making them potential candidates for biomedical uses [16]. The carbon tetrachloride soluble fraction of *Glycosmis arborea* stems contains a substantial amount of phenolic compounds, indicating possible antioxidant activity [17].

In recent years, free radicals along with their associated species have garnered a lot of interest. Their primary sources are oxygen (also known reactive oxygen species [ROS]) and nitrogen (also known as reactive nitrogen species [RNS]), which are produced in the human body through a number of endogenous mechanisms, exposure to different physicochemical situations, or pathological states [12, 18]. There are three ways that free radicals can form: (a) by hemolytically breaking a normal molecule’s covalent bond; (b) by removing an electron from a regular molecule; (c) by means of an electron’s attachment to a regular molecule [19].

During regular cellular metabolism, ROS are produced in living cells and can damage important macromolecules such as proteins, lipids, carbohydrates, and nucleic acids. An imbalance between ROS and protective antioxidants leads to oxidative stress. Numerous diseases are caused by oxidative stress, which disrupts a number of cellular processes and results in a variety of pathological conditions where ROS overpower the body’s antioxidative defenses. These conditions include tissue damage, accelerated cellular death, and oxidative modification of the biological macromolecules mentioned above [20].

Antioxidants are chemical agents that either directly scavenge ROS, indirectly increase antioxidant defenses, or inhibit the generation of ROS, thus reducing oxidative stress [20, 21]. Although synthetically produced antioxidants, such as butylated hydroxytoluene (BHT), propyl gallate (PG), butylated hydroxyanisole (BHA), and others, have been used for decades, recent research has demonstrated that their use is unsafe due to their long‐term negative effects, which include ischemia/reperfusion injury, cardiovascular disease, atherosclerosis, tissue damage, and carcinogenesis [21]. As a result, research into safer antioxidants from natural sources, e.g., indigenous medicinal plants, is growing daily.

Although antioxidant activities have been reported for other *Glycosmis* species, the antioxidant potential of *G. cyanocarpa* has not been well studied. To date, there is no extensive study of *G. cyanocarpa* employing multiple in vitro antioxidant assays together with the quantification of total phenolic content (TPC) and total flavonoid content (TFC). This research gap makes it a valuable candidate for comprehensive antioxidant study and correlation analysis. The current study aimed to determine the TPC and TFC of the crude MeOH extract of *G. cyanocarpa* (GCM), evaluate its free radical scavenging activity using DPPH, ABTS, and FRAP assays, and assess the associations between TPC, TFC, and antioxidant activities using Pearson’s correlation analysis.

## 2. Materials and Method

### 2.1. Collection of Sample and Preparation

In July 2019, the stem and leaf of *G. cyanocarpa* were collected from Narshingdi, Bangladesh. The plant was accurately recognized by a specialist from the Bangladesh National Herbarium (BNH), and a specimen voucher was filed for future use with this collection, bearing accession number 48257. All parts of the plant were cleaned thoroughly. After being divided into little pieces, the plant components were left to dry for 7 days in the shade. Then, using an advanced grinding machine, the dry material was meticulously crushed into a coarse powder. The finished product weighed 1.5 kg and was used as the sample.

### 2.2. Extraction

For a period of 2 weeks, 1.5 kg of air‐dried powdered sample of *G. cyanocarpa* was stored immersed in 10 L of distilled methanol (MeOH) in a clean, amber bottle, which was shaken and stirred occasionally. Following an initial 2‐week cold extraction, the resulting mixture was filtered through a wide funnel using a cotton plug and a Buchi Rotavapour (Essen, Germany) was used to reduce the filtrate volume. This extraction process was repeated three additional times with fresh solvent over a span of 6 days, and the same beaker was used to collect the extracts. The dried methanol extract yielded in the end was 42 g (2.8%).

### 2.3. TPC

The modified Folin–Ciocalteu assay protocol was used to determine TPC [22]. Each *G. cyanocarpa* crude MeOH (GCM) extract (1 mg/mL) was mixed with 1 mL of 1 N Folin–Ciocalteu reagent and incubated for 5 min. The mixture was combined with 2 mL of 20% (w/v) Na_2_CO_3_. The mixture was centrifuged for 1 min at 15,000 g after 10 min at room temperature, and the absorbance at 765 nm was measured from the supernatant by a spectrophotometer using methanol as a blank (Libra S22, Biochrom Ltd., Cambridge, United Kingdom). The standard calibration curve was constructed using gallic acid solutions at concentrations of 25, 50, 75, and 100 *μ*g/mL, prepared by diluting a 1‐mg/mL stock solution in methanol. TPC concentration of the extract was calculated and recorded in milligrams of gallic acid equivalents (mg GAE/g extract).

### 2.4. TFC

The extract’s TFC was ascertained by employing the aluminum chloride colorimetric method [17, 23]. A calibration curve was constructed using concentrations of 25, 50, 75, and 100 *μ*g/mL obtained by diluting a 1 mg/mL standard quercetin stock solution with methanol. A methanolic extract solution (1 mg/mL) was also prepared. About 0.5 mL of every standard and sample concentration was mixed with 0.1 mL 10% AlCl_3_, 0.1 mL 1 M potassium acetate, and 4.3 mL distilled water. The blend was incubated at room temperature for 30 min. Methanol was used as a blank to verify the absorbance at 415 nm. Three replicates for every experiment were performed, and mg QE/g extract, or milligrams of quercetin equivalent per gram of extract, was used to denote the TFC.

### 2.5. DPPH Free Radical Scavenging Assay

In order to examine the capacity of GCM to scavenge 1, 1‐diphenyl‐2‐picrylhydrazyl free radicals, 3.0 mL of a methanol solution of DPPH (20 *μ*g/mL) was combined with 2.0 mL of methanolic extract solution in different successively diluted concentrations (500–0.98 *μ*g/mL). After incubation in the dark for 1 h to allow sufficient reaction time for slower‐reacting antioxidant compounds present in the plant extract [9], the absorbance at 517 nm was assessed via UV spectrophotometry, with methanol as the blank, and methanolic DPPH solution as the control (Libra S22, Biochrom Ltd.). Percent inhibition (%I) was calculated by the use of Equation (1). Antioxidant activity was identified through the comparison of IC_50_ value of GCM with ASA (ascorbic acid) [9, 22].

(1) %inhibition%I=Abs Control−Abs Sample/Abs Control

### 2.6. ABTS Radical Scavenging Assay

In order to compare the GCM extracts’ capacity to scavenge free radicals, the ABTS radical cation (ABTS+˙) decolorization assay [24] was also carried out with minor modifications. The assay is based on the antioxidants of the extract reducing ABTS+˙ radicals. A 7‐mM concentration of ABTS was obtained by dissolving it in deionized water. The ABTS solution was reacted with 2.45 mM potassium persulfate to generate the ABTS+˙ radicals, which was then allowed to sit at room temperature for 12–16 h in the dark. For the study, deionized water was used to dilute the ABTS+˙ solution until it had an absorbance of 0.7 (±0.02) at 734 nm. At 10 min after the initial mixing of 3 mL of ABTS+˙ solution with 100 *μ*L of methanolic GCM extract solutions (10–100 *μ*g/mL), absorbance was recorded at 30°C using a UV‐Vis spectrophotometer at 734 nm with methanol as the blank. A control with 100 *μ*L methanol and 3 mL ABTS+˙ solution was also prepared for comparison. All assays were performed in triplicate, and each solution was used on the day of preparation. From the above equation (Equation (1)), the ABTS+˙ inhibition percentage was determined. Following that, the IC_50_ of ASA and test sample (GCM) was compared.

### 2.7. Ferric Reducing Antioxidant Power (FRAP) Assay

The ferric reducing ability of plant extracts was assessed by employing a modified FRAP assay [24]. This technique relies on antioxidants that contribute electrons, which, at low pH, cause a colorless ferric complex (Fe^3+^‐tripyridyltriazine) to be reduced to a blue colored ferrous complex (Fe^2+^‐tripyridyltriazine). To monitor the increase, the absorbance change at 593 nm is measured. On the day of the experiment, 10 mL of acetate buffer (300 mM, pH 3.6), 1 mL of 10 mM TPTZ (2,4,6‐tri(2‐pyridyl)‐s‐triazine) in 40 mM hydrochloric acid, and 1 mL of ferric chloride (20 mM) were combined to obtain the functional FRAP reagent. Using the freshly prepared FeSO_4_ at different concentrations (0.1–1 mM), a standard curve was created. Three milliliters of freshly made FRAP reagent was combined with 100 *μ*L of sample solutions and 300 *μ*L of deionized water. The mixture was kept in incubation for 30 min in a water bath at 37°C. The samples’ absorbance was then determined at 593 nm. Additionally, an acetate buffer was used to obtain a blank reading. By calculating the difference between the absorbance of the sample and the absorbance of the blank, the FRAP value was ascertained. Every measurement was carried out three times. The reducing capacity of the plant extracts under test was determined in this assay using the reactive signal provided by the Fe^2+^ solution. FRAP values were presented as mmol Fe^2+^ Eq/g of extract.

### 2.8. Statistical Analysis

Experiments were conducted in triplicate using independently prepared technical replicates from the same crude extract. Data obtained from the study were analyzed using SPSS 27.0 and the obtained results are represented as mean ± SD. Using one‐way ANOVA followed by Dunnett’s post hoc test for multiple comparisons against the control, statistical significance was evaluated, at various probability levels ( ^∗^ *p* < 0.05,  ^∗∗^ *p* < 0.01,  ^∗∗∗^ *p* < 0.001). Furthermore, Pearson’s correlation coefficients were employed to assess the associations between TFC, TPC, and in vitro antioxidant activities (DPPH, ABTS, and FRAP assays) of the crude GCM.

## 3. Results

### 3.1. TPC

**Figure 1:**
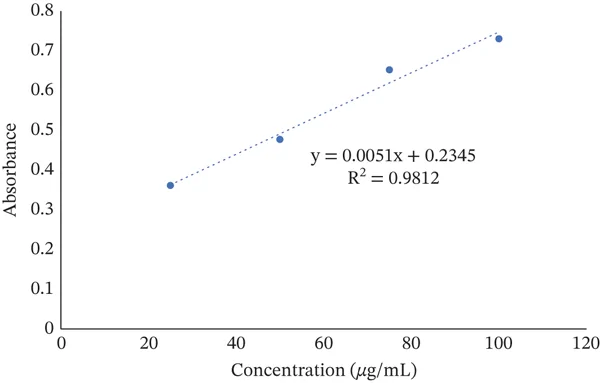
Standard curve of gallic acid for TPC.

Gallic acid was employed as the standard in the Folin–Ciocalteu assay for determining the TPC of GCM. The content was estimated based on the regression equation (y = 0.0051x + 0.2345, *R* ^2^ = 0.9812) of the calibration curve, indicating a strong correlation between concentration and absorbance (Figure 1). The phenolic content of the extract was 44.84 ± 0.99 mg gallic acid equivalent (GAE) per gram of dry extract (Table 1).

**Table 1:** Total phenolic content [mg gallic acid equivalent (GAE) per gram dry extract] and total flavonoid content [mg quercetin equivalent (QE) per gram dry extract] of GCM.

GCM	*M* *e* *a* *n* ± *S* *D*
TPC	44.84 ± 0.99 mg GAE/g extract
TFC	60.83 ± 2.60 mg QE/g extract

### 3.2. TFC

**Figure 2:**
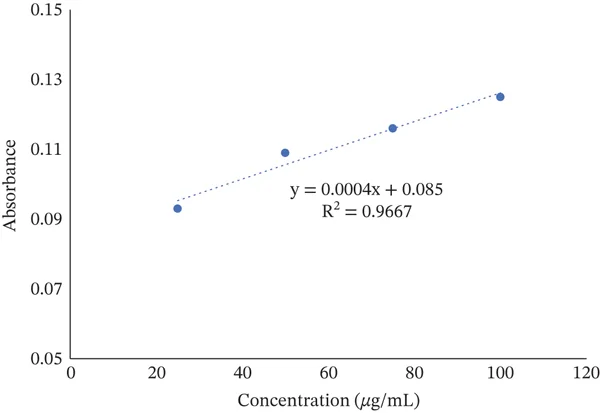
Standard curve of quercetin for TFC.

To quantify the TFC of the crude GCM, a calibration curve was constructed where quercetin was used as the reference standard (Figure 2). The resulting linear equation y = 0.0004x + 0.085, had a high coefficient of determination (*R* ^2^ = 0.9667), suggesting that concentration and absorbance were strongly correlated. Based on the standard curve, the plant extract was found to contain a flavonoid content of 60.83 ± 2.60 mg quercetin equivalent (QE) per gram of dry extract (Table 1).

### 3.3. DPPH Free Radical Scavenging Assay

**Figure 3:**
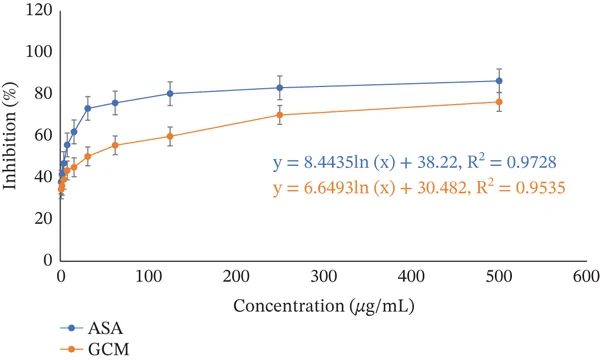
% inhibition vs concentration curve of GCM and ASA in DPPH assay. Values represent mean ± standard deviation of triplicate experiments (*n* = 3). Error bars indicate SD at each concentration.

The DPPH free radical scavenging activity of GCM is illustrated in Figure 3 and Table 2. The extract demonstrated significant (*p* < 0.001) free radical inhibition, with an IC_50_ value of 18.80 ± 0.42 *μ*g/mL, indicating strong antioxidant potential. In comparison, the standard ASA exhibited a lower IC_50_ value of 4.06 ± 0.13 *μ*g/mL, reflecting its potent scavenging activity. Notably, GCM showed statistically significant (*p* < 0.001) percent inhibition at all tested concentrations relative to the control. These results imply that *G. cyanocarpa* has potential as a source for the discovery of novel antioxidant compounds.

**Table 2:** IC_50_ values of ascorbic acid and crude MeOH extract of *G. cyanocarpa* in DPPH assay and ABTS assay.

Sample code	IC_50_ (*μ*g/mL) (*m* *e* *a* *n* ± *S* *D*)	
	In DPPH assay	In ABTS assay
GCM	18.80 ± 0.42	74.54 ± 0.31
Standard (ASA)	4.06 ± 0.13	17.25 ± 0.16

### 3.4. ABTS Radical Scavenging Assay

**Figure 4:**
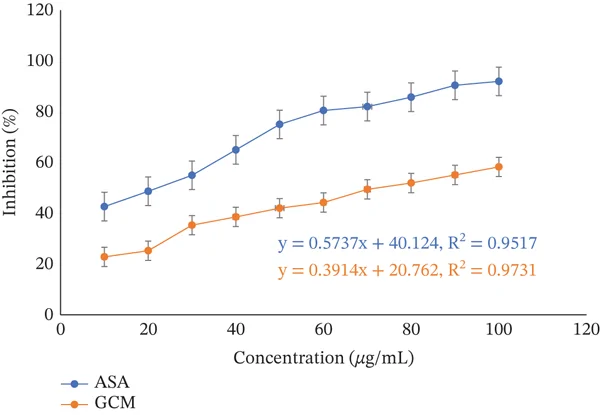
% inhibition vs concentration curve of GCM and ASA in ABTS assay. Values represent mean ± standard deviation of triplicate experiments (*n* = 3). Error bars indicate SD at each concentration.

In the ABTS assay, GCM exhibited notable free radical scavenging activity, with an IC_50_ value of 74.54 ± 0.31 *μ*g/mL (Table 2). In contrast, the standard ASA demonstrated higher antioxidant activity, with a lower IC_50_ value of 17.25 ± 0.16 *μ*g/mL. The percent inhibition observed for GCM was statistically significant (*p* < 0.001) across all tested concentrations (Figure 4). Further research focusing on the purification of GCM may facilitate its development as a prospective source of natural antioxidants.

### 3.5. Ferric Reducing Antioxidant Power (FRAP) Assay

**Figure 5:**
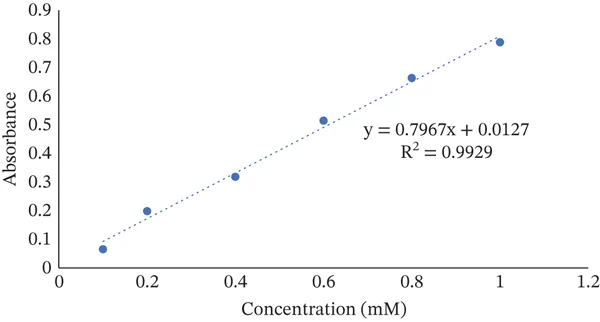
Standard curve of ferrous sulfate for FRAP assay.

The ferric reducing activity of the GCM was estimated using a standard curve of ferrous sulfate as a reference standard (Figure 5). The linear equation obtained was y = 0.7967x + 0.0127, with a high coefficient of determination (*R* ^2^) of 0.9929. This strongly implies that the concentration and absorbance measurements fit well together. Based on the calibration curve, GCM exhibited a ferric reducing capacity of 2.42 ± 0.33 mmol Fe^2+^ equivalent per gram dry extract (Table 3).

**Table 3:** Ferric reducing antioxidant power (FRAP) [mmol Fe^2+^ equivalent per gram dry extract] of GCM.

GCM	*M* *e* *a* *n* ± *S* *D*
FRAP assay	2.42 ± 0.33 mmol Fe^2+^ Eq/g dry extract

### 3.6. Pearson’s Correlation Coefficient (r) Between Antioxidant Assays

Pearson’s correlation analysis was performed to analyze the associations among the TPC, TFC, and antioxidant activities exerted by DPPH, ABTS, and FRAP assays (Table 4). The findings indicated a strong positive correlation between TPC and DPPH (r = 0.998, *p* < 0.05), suggesting that phenolic compounds are associated with free radical scavenging effect. TFC also revealed a high correlation with FRAP (r = 0.999, *p* < 0.05), indicating that flavonoids are significantly associated with reducing power. Additionally, strong correlations were identified between ABTS and both TPC (r = 0.957) and TFC (r = 0.977), implying an association between these phytochemical constituents and the overall antioxidant potential. Collectively, these findings highlight that phenolic and flavonoid contents are associated with antioxidant activity across multiple assay methods; however, these correlations are not causative, they are statistical associations.

**Table 4:** Pearson’s correlation coefficient (r) between antioxidant assays.

Pearson’s correlation coefficients (r)					
	TPC	TFC	DPPH	ABTS	FRAP
TPC	1				
TFC	0.874	1			
DPPH	0.998 ^∗^	0.899	1		
ABTS	0.957	0.977	0.971	1	
FRAP	0.847	0.999 ^∗^	0.875	0.965	1

^∗^p <0.05.

## 4. Discussion

Antioxidants exhibit their activity in various mechanisms, such as inhibiting free radical generation, scavenging free radicals, and demonstrating reducing power; hence, it is not possible to precisely characterize every individual antioxidant in a complex or mixed system for a single assay [25]. So, to assess the antioxidant capacities of compounds, it is crucial to employ at least two distinct techniques [26].

Three antioxidant assays—DPPH (2,2‐diphenyl‐1‐picrylhydrazyl), ABTS (2,2 ^′^‐azino‐bis(3‐ethylbenzothiazoline‐6‐sulfonic acid)) and ferric reducing antioxidant power (FRAP)—were employed in this study for assessing the antioxidant properties of GCM extracts. The contents of secondary metabolites, including TPC and TFC, were also measured.

The phenolic content of GCM was relatively high, as it was estimated to be 44.84 ± 0.99 mg GAE per gram of dry extract (Table 1). It indicated that *G. cyanocarpa* may be a good source of phenolic compounds. The result was obtained using a calibration curve with good linearity (*R* ^2^ = 0.9812) (Figure 1).

With a wide range of structural changes and chemical compositions, phenolic compounds are effective natural antioxidants. Phenolic acid, flavonoids, and tocopherols are the three most important natural antioxidant groups [20]. Previous studies reported that phenolic compounds such as 1,2‐Benzenediol,4‐(2‐aminopropyl)‐, and Phenol, 2,4‐bis(1,1‐dimethylethyl)‐, phosphite (3:1) are isolated from *G. cyanocarpa* [27]. Also scientists had isolated and characterized another novel phenolic compound, 4‐(3‐hydroxy‐2‐methylpropyl)‐2‐methoxyphenol, from this plant [9]. Therefore, the presence of high TPC may be a major contributor to the antioxidant property of GCM.

The flavonoid content of GCM was also found to be significantly high, with a value of 60.83 ± 2.60 mg quercetin equivalent per gram of dry extract (Table 1). It suggests a considerable presence of flavonoid compounds in *G. cyanocarpa*. The calibration curve used for quantification has a high coefficient of determination (*R* ^2^ = 0.9667), indicating strong linearity (Figure 2).

In the DPPH assay, the extract displayed excellent antioxidant capacity with an IC_50_ value of 18.80 ± 0.42 *μ*g/mL (Table 2). All the tested concentrations demonstrated significant (*p* < 0.001) free radical inhibition, indicating efficient and strong antioxidant activity. Even though, when compared with ASA, which had a much lower IC_50_ value of 4.06 ± 0.13 *μ*g/mL, GCM seems less effective; its significant ability to scavenge free radicals, however, emphasizes its potential as a natural source of antioxidants.

Previous studies on *G. cyanocarpa* reported significant DPPH radical scavenging activity, particularly from the ethyl acetate fraction of stem and leaf extracts, which was comparable with ASA at 200 *μ*g/mL [7]. Additionally, a newly isolated phenolic compound, 4‐(3‐hydroxy‐2‐methylpropyl)‐2‐methoxyphenol, showed 92.96% activity, closely matching that of BHT (96.48%) [9]. The present study extends these findings by evaluating the crude extract’s antioxidant potential through five in vitro assays and analyzing inter‐assay correlations to provide a holistic understanding of the extract’s antioxidant profile.

As for the ABTS assay, GCM showed good free radical scavenging property with an IC_50_ value of 74.54 ± 0.31 *μ*g/mL; however when compared, ASA demonstrated greater antioxidant potency, indicated by its lower IC_50_ value of 17.25 ± 0.16 *μ*g/mL (Table 2).

In the FRAP assay, the ferric reducing activity of GCM was evaluated using a standard curve created with ferrous sulfate. The equation for this curve was y = 0.7967x + 0.0127, with a high *R* ^2^ value of 0.9929, which means the relationship between concentration and absorbance was very strong and reliable (Figure 5). Using this calibration curve, GCM was found to have a ferric reducing capacity of 2.42 ± 0.33 mmol Fe^2+^ equivalent per gram of dry extract (Table 3). This indicates that GCM has the ability to reduce ferric (Fe^3+^) ions to ferrous (Fe^2+^) ions, implying its antioxidant potential. A higher reducing capacity suggests stronger antioxidant activity, as reducing agents help neutralize harmful free radicals.

Pearson’s correlation analysis showed a strong association between TPC, TFC, and antioxidant activity as estimated by DPPH, ABTS, and FRAP assays (Table 4). The significant positive correlation between TPC and DPPH (r = 0.998, *p* < 0.05) indicates that higher phenolic content is associated with greater free radical scavenging activity. Similarly, the strong correlation between TFC and FRAP (r = 0.999, *p* < 0.05) suggests an association between flavonoid content and the reducing power of the extract.

Additionally, the strong positive correlations of ABTS with both TPC (r = 0.957) and TFC (r = 0.977) further support the association of phenolic and flavonoid contents with the antioxidant capacity of the plant. Similar positive correlations between phenolic composition and antioxidant activity were also reported in previous research [28–31]. These correlations, however, represent statistical associations rather than causal relationships, and further phytochemical and mechanistic investigations are needed to establish the particular contributions of these constituents to the observed antioxidant activity. Overall, the findings suggest that the plant is a good natural source of bioactive chemicals since flavonoids and phenolics have synergistic interactions that amplify its antioxidant activity [32, 33].

Previous studies on *Glycosmis* species have revealed substantial antioxidant capacity. In a prior investigation, the ethanolic leaf extract of *G. pentaphylla* exhibited potent free radical scavenging in DPPH (EC_50_ = 29.04 ± 0.008 *μ*g/mL) and ABTS (EC_50_ = 49.02 ± 0.006 *μ*g/mL) assays, along with high TFC (112.96 ± 3.89 mg QE/g extract) and TPC (96.6 ± 1.08 mg GAE/g extract) [15]. In another study, a significant amount of phenolic compounds (36.95 ± 0.54 mg GAE/g extract) was found in the carbon tetrachloride soluble fraction of *G. arborea* stems [17], implying that members of this genus are rich in antioxidant phytoconstituents. The present study identifies *G. cyanocarpa* as a promising antioxidant source through comprehensive in vitro assays and correlation analysis, demonstrating comparable antioxidant potential within the genus. These findings suggest its use as an antioxidant‐rich herbal resource; however, the study is limited to in vitro evaluation and does not encompass bioactivity‐guided active constituent isolation, mechanistic investigations or in vivo or cell‐based validation. The active compounds contributing the observed antioxidant activity and their mechanisms are yet to be identified. Future research should focus on isolating and characterizing the active constituents, followed by mechanistic and biological validation to confirm their therapeutic efficacy in oxidative stress‐related disorders.

## 5. Conclusion

It was revealed in the in vitro studies that MeOH crude extract from the leaves and stems of *G. cyanocarpa* has a vast amount of phenolic and flavonoid contents. The extract exhibited notable antioxidant activity in DPPH, ABTS, and FRAP assays, with Pearson’s correlation analysis proving high positive correlations between TPC, TFC, and antioxidant efficacies. All of these findings point out the antioxidant potential of *G. cyanocarpa*, highlighting it as a promising source of bioactive compounds and propounding further exploration of its active constituents and therapeutic applications. The discovered antioxidant activities suggest the potential use of this plant in the formulation of natural antioxidant‐based health products.

## Author Contributions

**Sania Ashrafi:** conceptualization, methodology, writing – review and editing, supervision, visualization, review and monitoring.

**Sadia Tasnim Mina:** investigation; data curation; software; formal analysis; writing – original draft, review and editing.

**Raihana Haque Jebin:** writing – original draft, review and editing.

**Firoj Ahmed:** resources; supervision.

## Funding

No funding was received for this manuscript.

## Conflicts of Interest

The authors declare no conflicts of interest.

## Data Availability

The data that support the findings of this study are available from the corresponding author upon reasonable request.
